# Supplementary material for: Generation of Flag/DYKDDDDK Epitope Tag Knock-In Mice Using i-GONAD Enables Detection of Endogenous CaMKIIα and β Proteins
Source: Int J Mol Sci. 2022 Oct 7;23(19):11915. doi: 10.3390/ijms231911915 (PMC9569722; doi:10.3390/ijms231911915)
Supplement: Supplementary file 1 [file ijms-23-11915-s001.zip › ijms-1906576-supplementary.pdf]

Supplementary Figure

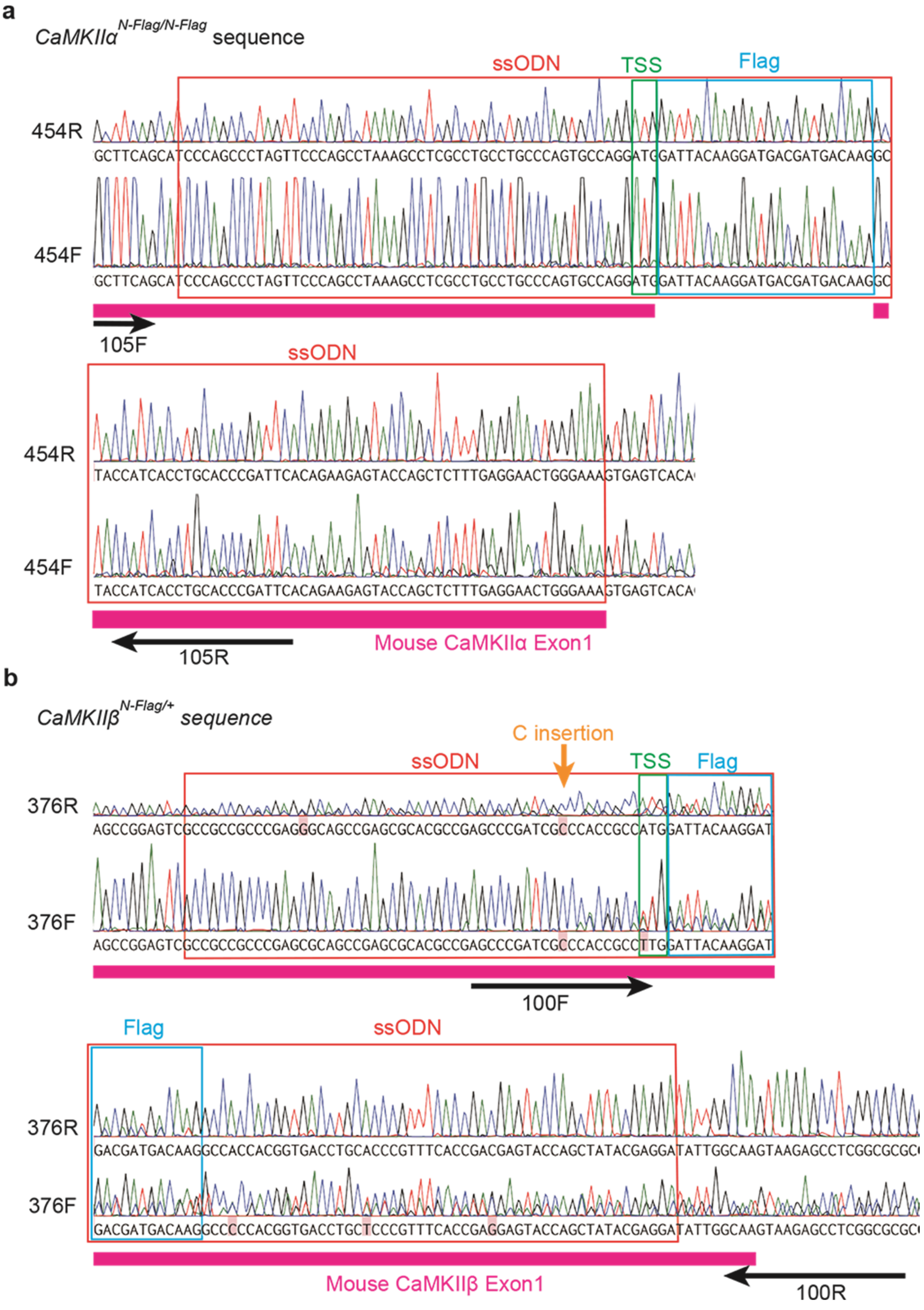

Figure S1. The Sanger sequence including ssODN of knock-in region of *CaMKIIα*$^{N\text{-}Flag/N\text{-}Flag}$

homozygous (**a**) and *CaMKIIβ*$^{N\text{-}Flag/+}$ heterozygous (**b**) mouse using 454F/454R primers for *CaMKIIα* and 376F/R primers for *CaMKIIβ*. Red square shows ssODN. Green square shows transcriptional starting site (TSS, ATG codon). Blue square shows Flag sequence. Pink bar shows exon1 region of mouse *CaMKIIα* and *CaMKIIβ* gene. Yellow arrow shows C insertion.
